# Bilious and Congestive Fever

**Published:** 1841-02

**Authors:** A. M. Keller

**Affiliations:** Alabama


BILIOUS AND CONGESTIVE FEVER. BY A. M. KELLER, M. D., OF ALABAMA.

We lay before our readers the following extract of a letter from a scientific physician of the South. Y.

In the treatment of a strictly bilious fever, purgatives are our great reliance; and we need not ransack the materia medica for a variety. Calomel, uncombined, is the mildest and most effectual—to be repeated as long as bilious evacuations appear. In long continued cases, particularly where there is no sensible remission, where calomel has been freely given, I have been in the habit of combining quinine and calomel together—3 grs. quinine, 4 of calomel—given every two hours. I look upon quinine as the most powerful sudorific in the whole catalogue of medicines. In large doses it acts as a sedative; for in numerous instances, I have seen the pulse reduced to a natural state in a few hours by its use. J. H. had been sick twelve or fourteen days with bilious fever, during the last eight of which, no sensible remission had taken place. He had been freely operated upon by mercurial purgatives, given both in full and broken doses, without any abatement of his fever; when, becoming alarmed for his safety, I commenced the above combination of calomel and quinine. When the first dose was given, his pulse was about 100 per minute; by the time he had taken the third dose it was at 75, the natural standard with him, and the whole surface was bathed in profuse perspiration, so much so, that it became unpleasant to the patient, and he strongly insisted on the windows being opened, on fanning, &c. By the time he had taken some thirty grains of the quinine, I thought it prudent to wait for the operation of the bowels, which soon took place effectually, and he had no more fever. The quinine was continued alone for several days. I look upon it as the only febrifuge we possess, strictly so called.

*Congestive Fever.*—Much has been said and written about this truly formidable disease. It is so insidious in its attack, and puts on, when it does approach, so exactly the form of chills and fever, that one who has never seen it would treat it as such; and would not, in all probability, discover his mistake until the disease had gone beyond his reach. It may be known by the greater severity and permanency of the chill, and a death-like coldness and lividity of the whole surface; great derangement of the sensorial functions, almost from the commencement. The tongue exhibits a most peculiar appearance—generally very little coated but very moist, and of a *purplish colour*; this last I consider almost a certain diagnostic of the disease. It generally, if fatal, terminates with the third chill; this generally happens about the fourth day. Sometimes I have known it to be constituted of a rapid succession of chills, destroying the patient in forty-eight hours. When called to the patient you must use no half-way remedies. Give calomel in large doses every two hours, until it operates on the bowels; if the discharges are not dark, pitchy, and consistent, check them at once by an opiate injection and re-commence the calomel in increased doses, and never stop until the discharges are right. To relieve the intense suffering of the patient from cold, give him hot and strong French brandy toddy ad libitum. If, notwithstanding your efforts, collapse supervenes, put your patient into a strong mustard bath; if that fails to excite the pulse, there is a last remedy you may resort to—what in medicine may truly be called a hydrostatic paradox. Strip your patient entirely, and dash about fifty gallons of cold water over him as hard and fast as you can; wipe hastily with warm towels and wrap him in hot blankets, and use any other exciting measures, as friction to the feet and hands. Make a black liniment as follows:

Olive oil ℥iss.
Spts. turpentine ℥iss.
Sulph. acid ʒiii.

Mix in an open vessel—to be rubbed on warm.

If the pulse revives, keep it up if you can with brandy; if it sinks again or does not rise from the first cold dash, try it again; and as long as your patient has life, for if this does not save him nothing will. I have seen it tried often, and in two-thirds of the cases it proved entirely successful. One case, I recollect, of a favorite servant; the family had despaired of her life, and had removed her to an adjoining room to die. She was entirely pulseless, and had been so for hours; the cold dash was applied twice in an hour, and in three hours the excitement was so great as to require the free use of the lancet; in a few days she was as well as ever. If cold water fails to excite in this last stage, you may consider the case of your patient hopeless.

*January*, 1841.

